# Association between hemoglobin glycation index and the risk of cardiovascular disease in early-stage cardiovascular-kidney-metabolic syndrome: evidence from the China health and retirement longitudinal study

**DOI:** 10.3389/fendo.2025.1554032

**Published:** 2025-05-08

**Authors:** Huiyi Liu, Shuai Mao, Yunzhang Zhao, Lisha Dong, Yifan Wang, Chao Lv, Tong Yin

**Affiliations:** ^1^ Institute of Geriatrics, Beijing Key Laboratory of Research on Comorbidity in the Elderly, National Clinical Research Center for Geriatric Diseases, Second Medical Center of Chinese PLA General Hospital, Beijing, China; ^2^ Medical School of Chinese PLA, Chinese PLA General Hospital, Beijing, China

**Keywords:** HGI, glycemic variability, cardiovascular kidney metabolic syndrome, CVD, CHARLS

## Abstract

**Background:**

Cardiovascular-kidney-metabolic (CKM) syndrome reflects the interplay among metabolic risk factors, chronic kidney disease, and cardiovascular disease (CVD). While the hemoglobin glycation index (HGI) has demonstrated prognostic value for cardiovascular events, its clinical utility remains unexplored in early-stage CKM syndrome.

**Methods:**

Participants with early-stage CKM syndrome (stage 0-3) were recruited from the China Health and Retirement Longitudinal Study (CHARLS) database. Using k-means clustering analysis, the participants were classified according to the values of HGI measured at baseline and 3 years later, respectively. The primary outcome was self-reported CVD during the follow-up of at least 3 years. Extreme gradient boosting (XGBoost) algorithm was applied, with the Shapley additive explanation (SHAP) method used to determine feature importance. Multivariable logistics proportional regression analysis the association between HGI and CVD, and restricted cubic spline (RCS) regression assessed potential nonlinear relationships.

**Results:**

A total of 4676 eligible participants were included in the final analysis, with 944 (20.19%) progressed to CVD within 10 years. Among the baseline clinical features, HGI ranked the second for the impact on the occurrence of CVD. According to the changes of HGI values, the participants were clustered into 4 classes. Compared to the class 1 with lower level of HGI, higher risk of CVD was observed in class 3 (adjusted OR: 1.34, 95% CI: 1.06-1.69, P = 0.013) and class 4 (adjusted OR: 1.65, 95% CI: 1.01-2.45, P = 0.025) with higher and rapidly increasing level of HGI. RCS analysis showed cumulative HGI and the risk of CVD were linearly related (P for nonlinearity = 0.967). Subgroup analyses confirmed the stability of the association. Additionally, the SHAP plot revealed that HGI were the more important features than traditional risk factors such as FBG for predicting CVD.

**Conclusion:**

HGI is associated with an elevated risk of CVD in participants with early-stage CKM syndrome. HGI can serve as an independent biomarker for guiding clinical decision-making and managing patient outcomes.

## 1 Introduction

Cardiovascular disease (CVD), chronic kidney disease (CKD), diabetes, and obesity are pathophysiologically interrelated that concurrently affect adult population with the prevalence of 5% in the United States (1), and 15% in China (2). In 2023, the American Heart Association defined this systemic condition as Cardiovascular-Kidney-Metabolic (CKM) syndrome, which may lead to premature mortality and increased morbidity (3). As reported, the combination of CKD and diabetes escalated the 10-year mortality rate markedly to 31.1% (4). The risk of CVD determines the staging and prognosis of CKM syndromes. Central to the CKM framework is the emphasis on risk-based primary prevention of CVD for individuals in CKM stages 0 to 3 (5). However, the prediction of the risk of CVD in the early stages of CKM syndromes is by far a challenge (6).

For patients with CKM syndromes, it is crucial to effectively control blood sugar levels and use reliable indicators to minimize diabetes-related complications and mortality (7). Glycosylated hemoglobin A1c (HbA1c) is strongly associated with the development of both microvascular and macrovascular diseases, likely due to its involvement in protein glycation (8). Despite standardized assays, discrepancies between HbA1c and other glycemic measures are well-documented and can affect the accuracy of glycemic control and management (9, 10). The mean erythrocyte lifespan, differences in cell membrane glucose transmembrane gradients and enzyme abnormalities can independently impact the reliability of HbA1c (11–13). Other genetic factors like genetic variation in hemoglobin can also affect the association of HbA1c with “true” average glucose exposure, particularly in the low (no diabetic) range (14). For most patients with metabolic disorders, a more tailored and individualized approach should be implemented to prevent vascular complications (10).

To solve these problems, the hemoglobin glycation index (HGI) was developed to directly reflect the individual glycemic variability by quantifying the difference between HbA1c and plasma glucose concentration (15). HGI could predict the risk of diabetic complications, including CVD, microvascular diseases, and mortality in patients with diabetes mellitus (16, 17). Presently, there have been few research that examined the correlation in patients with complex metabolic abnormalities. Prior research has shown that HGI was positively associated with the incidence of obesity, increased levels of low-density lipoprotein, triglyceride, and postprandial glycemic excursion, respectively (18). Therefore, in this study, we aimed to examine the association between HGI and the risk of CVD in the early stages of CKM syndromes.

## 2 Methods

### 2.1 Data source and study population

This prospective study used data from the China Health and Retirement Longitudinal Study (CHARLS), which includes clinical information from participants aged 45 years in China from 2011 to 2012 were considered as baseline (Wave 1), and follow-up each two years. Up to now, CHARLS has released three waves of follow-up data (Wave 2 in 2013, Wave 3 in 2015 and Wave 4 in 2018). The protocol of CHARLS study was approved by the Ethical Review Committee of Peking University (IRB00001052-11015). Informed consent was obtained in writing from all participants prior to their inclusion.

**Figure 1:**
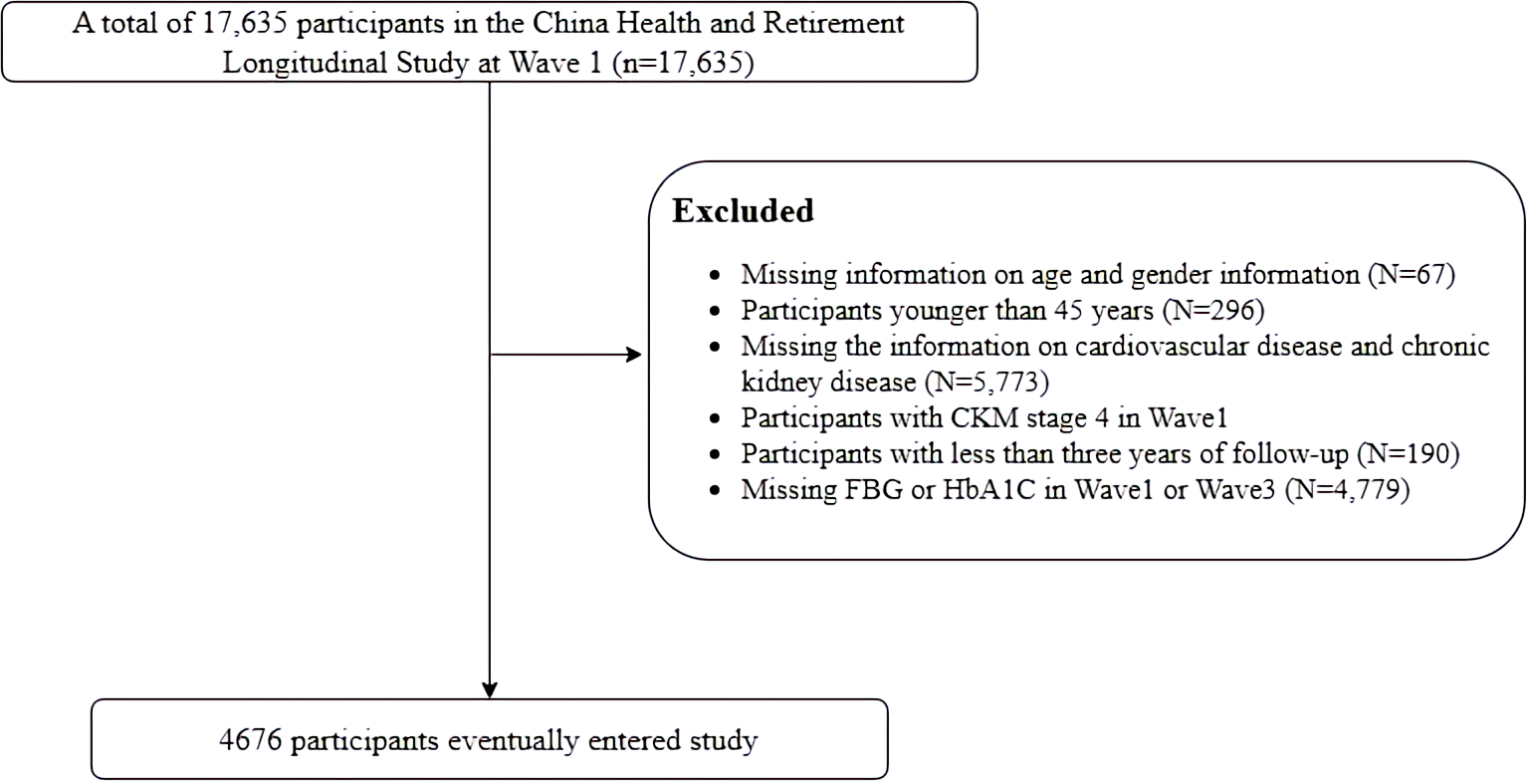
Flowchart of the study population.

In the study, 17,635 individuals who completed the baseline survey were included in the analysis. We excluded 12,959 individuals for the following reasons: (1) lack of information on age and gender, (2) participants younger than 45 years, (3) absence of information on cardiovascular disease and chronic kidney disease, (4) participants with CKM stage 4 at baseline, already diagnosed with CVD, (5) participants with less than 3 years of follow-up, (6) lack of data on FBG or HbA1c. Ultimately, a total of 4,676 individuals were included in the analysis (**Figure 1**).

### 2.2 Deriving HGI from the HbA1c versus FBG regression equation

HGI was calculated following the methodology established by Hempe et al. (19). To estimate the inter-individual variance in HbA1c levels, we utilized baseline FBG and HbA1c data for our calculations. The predicted HbA1c level was calculated for each participant through linear regression analysis (HbA1c = 0.017 × FBG + 3.41). HGI was then defined as the difference between the measured HbA1c and the predicted HbA1c (HGI = measured HbA1c – predicted HbA1c). Cumulative HGI was calculated using hematological data from Waves 1 and 3, derived with the formula: (HGI_2012_ + HGI_2015_)/2 × time.

### 2.3 Determination of endpoints

The primary outcome was the incidence of CVD, adhering to the previous protocols established with the CHARLS dataset. It was ascertained by the question “Did your doctor tell you that you have been diagnosed with a heart attack, angina pectoris, coronary heart disease, heart failure, or other heart problem?” (6).

### 2.4 Data collection and definitions

Baseline data regarding socio-demographic status and disease-related information were collected through in-person interviews conducted by trained interviewers using a structured questionnaire. The socio-demographic information gathered included age, gender, education, and marital status. Disease-related factors encompassed body mass index (BMI), systolic blood pressure (SBP), diastolic blood pressure (DBP), hypertension, dyslipidemia, diabetes, lifestyle factors (such as smoking and alcohol consumption). Participants were required to fast for at least 12 hours prior to the measurement of FBG, triglycerides (TG), total cholesterol (TC), high-density lipoprotein cholesterol (HDL-C), and low-density lipoprotein cholesterol (LDL-C). BMI was calculated as weight (kg) divided by height squared (m²). Participants were categorized into three groups based on their BMI: as normal weight (< 25 kg/m^2^), overweight (25–29.9 kg/m^2^), and obesity (≥ 30 kg/m^2^) (20).

Hypertension was defined as a history of diagnosis, the use of antihypertensive medication, systolic blood pressure ≥140 mmHg, or diastolic blood pressure ≥ 90 mmHg (21). Diabetes was defined as self-reported diagnosis history, use of any insulin or oral hypoglycemic agents, and fasting glucose ≥ 7.0 mmol/L or an HbA1c of ≥ 6.5% at baseline (22). Dyslipidemia was defined by lipid abnormalities, including TC ≥ 240 mmol/L, LDL-C >160 mmol/L, TG>150 mmol/L, or HDL-C < 40 mmol/L or use of any lipid-lowering treatment (23).

The classification of CKM syndrome, as outlined in the AHA Statement, provides a comprehensive framework for the early assessment of risk factors and disease progression. Stage 0 is defined as the absence of CKM syndrome risk factors in healthy subjects. Stage 1 is characterized by overweight, abdominal obesity (waist circumference ≥ 80 cm in women and ≥ 90 cm in men), or prediabetes. Stage 2 involves the presence of at least one metabolic risk factor (such as hypertriglyceridemia, dyslipidemia, hypertension, metabolic syndrome, diabetes) or CKD. Stage 3 encompasses subclinical cardiovascular disease with a high predicted CVD risk calculated using the Framingham risk score. The estimated glomerular filtration rate (eGFR) was calculated using the Chinese Modification of Diet in Renal Disease (C-MDRD) equation to classify renal function according to the Kidney Disease Improving Global Outcomes (KDIGO) (3).

### 2.5 Statistical analysis

**Figure 2:**
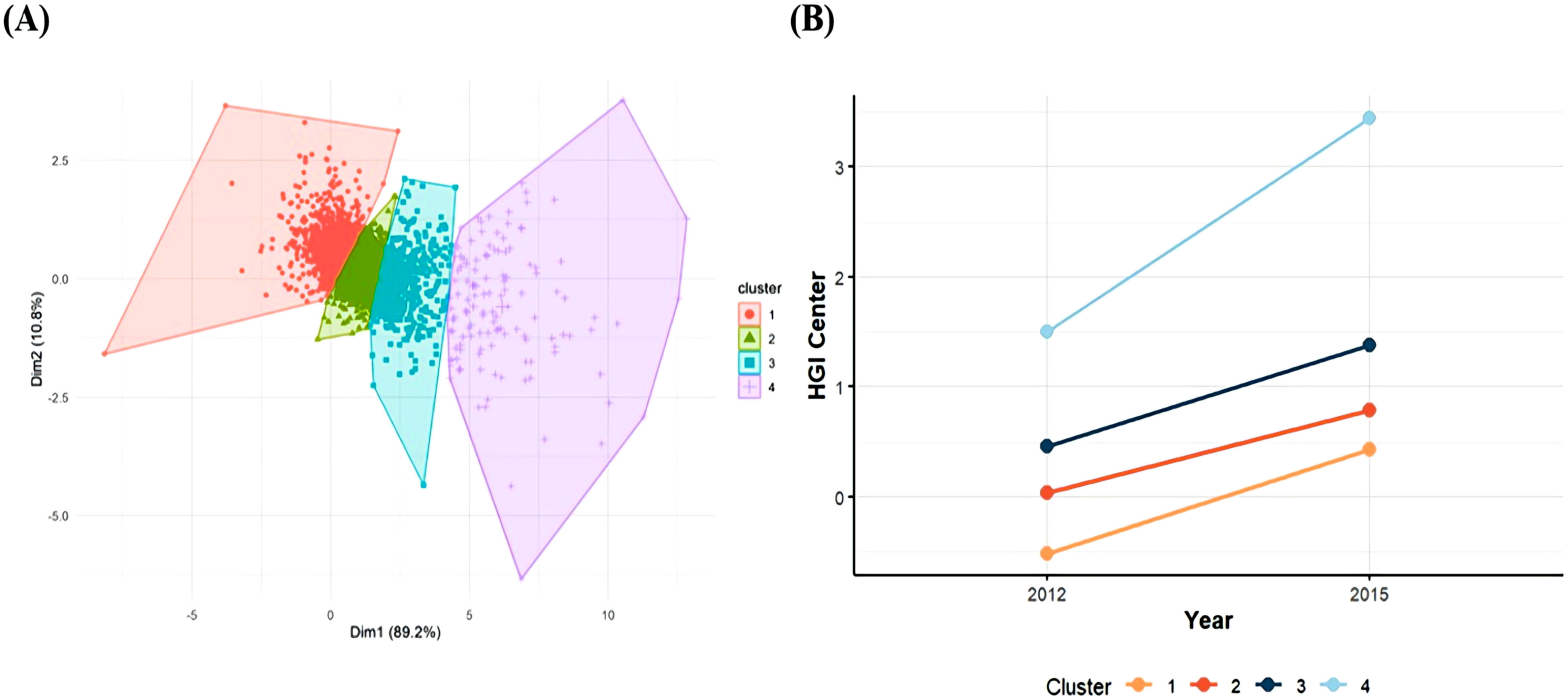
**(A)** The HGI clustering by k-means; **(B)** The trend of HGI changes in different clusters.

This study investigated HGI changes participants from Wave 1 and Wave 3 using k-means clustering within a logistic regression equation. K-means clustering, an unsupervised machine learning technique, groups data by minimizing distances within clusters, thereby partitioning the dataset into K distinct classes (24, 25). Each cluster was represented by a clustering center, defined as the mean value of all points within that cluster. The inflection point on the curve is considered the optimal number of clusters, representing the best division of the dataset. In our analysis, when K = 4, the curve tends to be steady (**Supplementary Figure S1**), so a four-cluster solution provided the optimal fit compared to other cluster counts (**Figure 2**). Data were presented as means ± standard deviation (SD) or median and interquartile range for continuous variables and percentages for categorical variables. Based on the result, we classified the participants into four groups: sustained low level (Class1); sustained medium level (Class2); high level and stable increasing (Class3); high level and fasting increasing (Class4). In Class 1 (n = 1305), the HGI ranged from -0.53 ± 0.39 in 2012 to 0.43 ± 0.46 in 2015, and the cumulative HGI was -0.10 ± 0.49, representing a consistently low and stable HGI; for Class 2 (n = 2334), the HGI ranged from 0.03 ± 0.25 in 2012 to 0.79 ± 0.31 in 2015, and the cumulative HGI was 0.82 ± 0.29, representing a sustained moderate HGI; for Class 3 (n = 902), the HGI ranged from 0.46 ± 0.44 in 2012 to 1.38 ± 0.49 in 2015, and the cumulative HGI was 1.83 ± 0.47, representing a high HGI with a slowly increasing trend (**Figure 2**). For Class 4 (n = 135), the HGI ranged from 1.51 ± 1.11 in 2012 to 3.46 ± 1.26 in 2015, and the cumulative HGI was 4.97 ± 1.36, representing a consistently high and fast increasing trend HGI.

Before investigating the association between HGI and incident CVD, we first employed machine learning algorithms for feature selection to determine their importance in the prognostic model (26). To improve the accuracy of feature selection, the eXtreme Gradient Boosting (XGBoost) model combined with SHapley Additive extension (SHAP) values was employed for feature screening and dimensionality reduction through Python package (version 3.11.10), identifying key features associated with CVD incident. The importance and the contribution of each feature can be directly observed by plotting the SHAP summary plot. Multiple imputations were used to fill in missing data to maximize statistical power and mitigate any bias that may result from missing data (27). Two logistic regression models were used to estimate the association between changes and cumulative measures in HGI with CVD, quantified through odds ratio (ORs) and 95% confidence intervals (CIs).

To investigate potential nonlinear associations between cumulative HGI and CVD events, we employed a RCS regression model with four knots. Interaction analyses were conducted to determine whether the association between cumulative HGI and CVD varied across covariates.

Mediation analysis was conducted to determine whether this association was mediated by risk factors, such as BMI and triglyceride glucose (TyG) index (28). Mediation analysis was performed using the ‘mediation’ package.

All statistical analyses were performed using R version 4.3.3 software (http://www.R-project.org/). Statistical significance was set as a two-sided p value < 0.05.

## 3 Result

### 3.1 Baseline characteristics of participants

In this study, a total of 4,676 participants were included for analysis. The mean age at baseline was 58.62 ± 8.65 years, with 2,170 participants (46.41%) identifying as men. The mean HGI was 0.00 ± 0.58 in 2012 and 0.88 ± 0.71 in 2015. **Table 1** presents the baseline characteristics of participants in each group based on the cluster analysis.

**Table 1:** Baseline characteristics of participants classified according to the changes of HGI.

Variables	Total (n = 4676)	Class 1 (n = 1305)	Class 2 (n = 2334)	Class 3 (n =902)	Class 4 (n = 135)	*P* value
Age, years	58.62 ± 8.65	57.84 ± 8.63	58.59 ± 8.77	59.82 ± 8.31	58.80 ± 7.97	<0.001
Gender, n (%)						<0.001
Male	2170 (46.41)	658 (50.42)	1096 (46.96)	363 (40.24)	53 (39.26)	
Female	2506 (53.59)	647 (49.58)	1238 (53.04)	539 (59.76)	82 (60.74)	
Education, n (%)						0.005
Middle school and above	1409 (30.13)	428 (32.80)	711 (30.46)	235 (26.05)	35 (25.93)	
No completion of middle school	3267 (69.87)	877 (67.20)	1623 (69.54)	667 (73.95)	100 (74.07)	
Smoking status, n (%)						0.051
Yes	1802 (38.54)	532 (40.77)	905 (38.77)	316 (35.03)	49 (36.30)	
No	2874 (61.46)	773 (59.23)	1429 (61.23)	586 (64.97)	86 (63.70)	
Drinking status, n (%)						<0.001
More than monthly	1211 (25.90)	383 (29.35)	619 (26.52)	187 (20.73)	22 (16.30)	
Less than monthly	393 (8.40)	127 (9.73)	186 (7.97)	66 (7.32)	14 (10.37)	
Never	3072 (65.70)	795 (60.92)	1529 (65.51)	649 (71.95)	99 (73.33)	
Hypertension, n (%)						<0.001
Yes	1700 (36.35)	493 (37.78)	781 (33.46)	347 (38.47)	79 (58.52)	
No	2976 (63.65)	812 (62.22)	1553 (66.54)	555 (61.53)	56 (41.48)	
Dyslipidemia, n (%)						<0.001
Yes	2214 (47.35)	630 (48.28)	1014 (43.44)	474 (52.55)	96 (71.11)	
No	2462 (52.65)	675 (51.72)	1320 (56.56)	428 (47.45)	39 (28.89)	
Diabetes, n (%)						<0.001
Yes	709 (15.16)	244 (18.70)	149 (6.38)	201 (22.28)	115 (85.19)	
No	3967 (84.84)	1061 (81.30)	2185 (93.62)	701 (77.72)	20 (14.81)	
BMI, kg/m^2^	23.55 ± 3.79	23.35 ± 3.61	23.40 ± 3.81	23.95 ± 3.86	25.71 ± 3.69	<0.001
SBP, mmHg	128.16 ± 41.18	129.84 ± 46.12	126.74 ± 37.18	126.46 ± 19.58	147.92 ± 106.47	<0.001
DBP, mmHg	75.83 ± 12.89	74.68 ± 12.39	75.98 ± 13.15	76.49 ± 12.95	79.98 ± 11.39	<0.001
TC, mg/dl	193.81 ± 38.27	189.95 ± 38.71	193.34 ± 37.57	198.72 ± 38.65	206.52 ± 37.85	<0.001
HDL-C, mg/dl	51.37 ± 15.44	50.65 ± 16.23	52.26 ± 14.95	50.96 ± 15.39	45.63 ± 14.57	<0.001
LDL-C, mg/dl	116.83 ± 34.89	110.31 ± 35.52	118.04 ± 33.98	121.46 ± 34.16	127.90 ± 38.95	<0.001
TG, mg/dl	130.62 ± 106.18	145.28 ± 146.64	119.29 ± 77.71	134.24 ± 95.96	160.64 ± 108.10	<0.001
BUN, mg/dl	15.59 ± 4.36	15.82 ± 4.38	15.52 ± 4.33	15.51 ± 4.35	15.17 ± 4.57	0.126
Scr, mg/dl	0.76 ± 0.17	0.77 ± 0.17	0.77 ± 0.17	0.76 ± 0.18	0.75 ± 0.19	0.253
Uric acid, mg/dl	4.38 ± 1.22	4.44 ± 1.25	4.37 ± 1.20	4.36 ± 1.21	4.11 ± 1.25	0.019
CRP, mg/dl	2.55 ± 6.86	2.20 ± 5.95	2.57 ± 6.71	3.01 ± 8.45	2.68 ± 5.44	0.056
WBC, ×10^9^/L	6.21 ± 1.88	6.02 ± 1.78	6.21 ± 1.88	6.41 ± 2.01	6.87 ± 1.74	<0.001
PLT, ×10^9^/L	212.68 ± 73.90	207.33 ± 72.44	210.87 ± 73.74	224.81 ± 75.94	214.77 ± 68.42	<0.001
HGI_2012_, %	0.00 ± 0.58	-0.53 ± 0.39	0.03 ± 0.25	0.46 ± 0.44	1.51 ± 1.11	<0.001
HGI_2015_, %	0.88 ± 0.71	0.43 ± 0.46	0.79 ± 0.31	1.38 ± 0.49	3.46 ± 1.26	<0.001
Cumulative HGI, %	0.88 ± 1.06	-0.10 ± 0.49	0.82 ± 0.29	1.83 ± 0.47	4.97 ± 1.36	<0.001
CVD, n (%)	716 (15.31)	184 (14.10)	331 (14.18)	171 (18.96)	30 (22.22)	<0.001

SBP: systolic blood pressure; DBP: diastolic blood pressure; BMI: body mass index; BUN: blood urea nitrogen; FBG: fasting blood glucose; TC: total cholesterol ; TG: triglyceride; HDL-C: high-density lipoprotein cholesterol; LDL-C: low-density lipoprotein cholesterol; CRP: C-reactive protein; HbA1c: hemoglobin A1C; UA: uric acid; Scr: serum creatinine; WBC: white blood cell count; PLT: platelet count; HGI: hemoglobin glycation index; CVD: cardiovascular disease

Notes: Continuous variables were expressed as mean ± standard deviation (SD) in case of normal distribution and compared between two groups by Kruskal-Wallis rank sum test. If the count variable had a theoretical number < 10, Fisher’s exact probability test was used. Categorical variables are presented as counts (percentages) and compared by Chi-square test.

Participants with higher and rapidly increasing levels of HGI were more likely to be female, older, and obese. Those also exhibited a greater prevalence of metabolic disorders, with significantly elevated levels of serum creatinine (Scr), serum uric acid (UA), TC, LDL-C, FBG, and HbA1c compared to participants in the lower-level clusters.

### 3.2 Association between the changes of HGI and CVD risk

**Figure 3:**
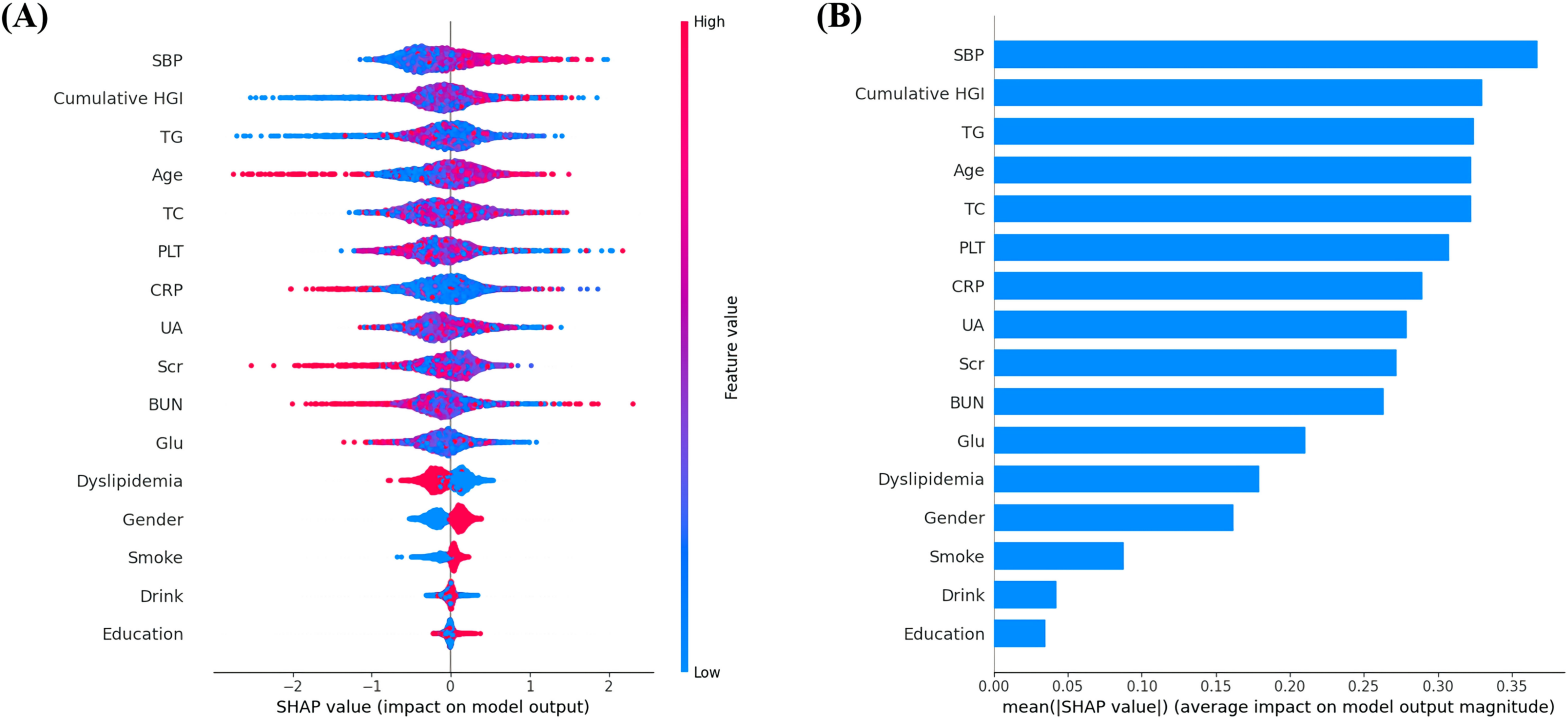
**(A)** Featured clinical variables screened by SHAP calculated by XGBoost model. Distribution of the impact of each feature on the model output. Each dot represents a patient in a row. The colors of the dots represent the feature values: red represents larger values and blue represents lower values. **(B)** The vertical axis shows the name of each variable, whereas the horizontal axis represents the feature value of each variable.

The incidence of 944 CVD during the follow-up period was presented in **Table 2**. The regression models were developed based on clinical expertise and feature importance selection results from the XGBoost algorithms, as shown in **Figure 3**. After adjusting for age, gender, smoking status, drink status, education, SBP, TG, TC, UA, platelet (PLT), Scr, blood urea nitrogen(BUN), FBG, and C-reactive protein (CRP) in Model 2, the multivariate-adjusted OR and 95% CI from lowest stable group to highest rapid increasing group were1.00 (reference), 0.97 (0.80, 1.18), 1.32 (1.05, 1.67), and 1.63 (1.01, 2.64), respectively.

**Table 2:** Association between HGI and the risk of CVD in patients with early-stage CKM syndrome.

Cluster of HGI	Crude	Model 1	Model 2
	OR (95% CI) *P* value	OR (95% CI) *P* value	OR (95% CI) *P* value
Class 1	Reference	Reference	Reference
Class 2	1.01 (0.83,1.22) 0.946	0.98 (0.81,1.20) 0.877	1.00 (0.82,1.22) 0.992
Class 3	1.43 (1.13,1.79) 0.002	1.34 (1.06,1.69) 0.013	1.35 (1.07,1.70) 0.012
Class 4	1.74 (1.13,2.69) 0.012	1.65 (1.06,2.55) 0.025	1.57 (1.01,2.45) 0.045

Model I, adjusted for age, gender.

Model II, adjusted for important feature calculated by XGBoost algorithm including: gender, age, education, smoking status, drink status, SBP, UA, Scr, TC, TG, BUN, PLT, FBG, CRP.

OR, odd ratio; CI, confidence interval.

### 3.3 Association between the value of cumulative HGI and CVD risk

Multivariable logistic regression analyses also indicated a positive relationship between the cumulative HGI and CVD risk, with an adjusted OR of 1.08 (95% CI: 1.04, 1.13) (**Table 2**). RCS regression analysis further confirmed the linear increase in CVD risk associated with higher values of the cumulative HGI (P for nonlinearity = 0.967, **Supplementary Figure S2**).

**Figure 4:**
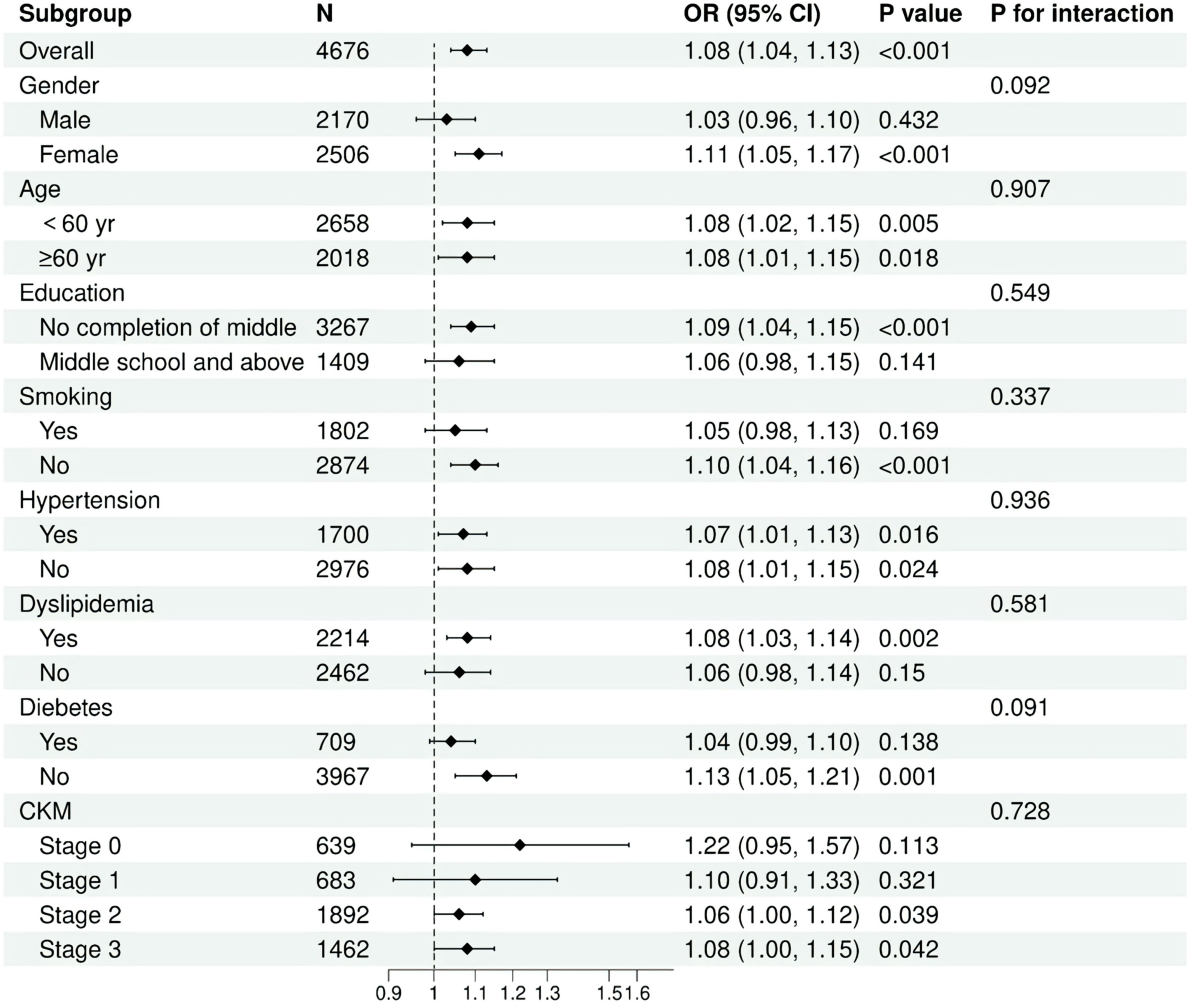
Forest plot of association between HGI and CVD in different subgroups.

Subgroup analyses and interaction tests were conducted to evaluate the consistency of the association between cumulative HGI and the risk of CVD across various individual subgroups, including age, gender, smoking status, hypertension, diabetes, dyslipidemia, and stage of CKM. Interaction terms were utilized to assess heterogeneity within each subgroup. No statistically significant interactions were identified regarding the association between cumulative HGI and CVD (**Figure 4** and **Supplementary Table S1**).

Overall, our results indicated that the positive association between cumulative HGI and CVD risk remains consistent across different population subgroups and is applicable in various settings.

### 3.4 Mediation analysis

In mediation analysis, it was suggested that BMI partially mediates the relationship between the cumulative HGI index and CVD, accounting for approximately 18.6% of the effect. The mediating role of the TyG index in the association was 6.9%. Dyslipidemia also played a significant role in mediating the association between the HGI index and new-onset CVD, contributing approximately 11.0% to the effect. However, diabetes, hypertension, and the chronic inflammation biomarker CRP did not demonstrate a significant mediating effect (**Table 3**).

**Table 3:** Mediation analysis for the associations between Cumulative HGI and CVD in patients with early-stage CKM syndrome.

Independent variable	Mediator	Total effect		Indirect effect		Direct effect		Proportion mediated, % (95% CI)
		Coefficient (95% CI)	*P* value	Coefficient (95% CI)	*P* value	Coefficient (95% CI)	*P* value	
Cumulative HGI	BMI	0.009 (0.003, 0.015)	0.016	0.002 (0.001, 0.003)	<0.001	0.007 (0.001, 0.013)	0.020	18.6 (8.0, 54.6)
Cumulative HGI	TyG	0.010 (0.004, 0.015)	<0.001	0.001 (0.000, 0.001)	0.036	0.009 (0.004, 0.015)	<0.001	6.9 (0.4, 19.8)
Cumulative HGI	Dyslipidemia	0.010 (0.004, 0.016)	<0.001	0.001 (0.000, 0.002)	0.032	0.009 (0.004, 0.015)	<0.001	7.8 (0.6, 26.7)
Cumulative HGI	CRP	-0.011 (-0.017, -0.006)	<0.001	0.001 (-0.001, 0.001)	0.380	-0.011 (-0.017, -0.006)	<0.001	5.0 (-0.7, 2.9)
Cumulative HGI	Hypertension	0.010 (0.004, 0.016)	<0.001	0.001 (-0.000, 0.002)	0.128	0.009 (0.003, 0.015)	<0.001	10.7 (-3.0, 28.1)
Cumulative HGI	Diabetes	0.010 (0.004, 0.016)	<0.001	0.000 (-0.001, 0.001)	0.608	0.010 (0.004, 0.016)	<0.001	2.4 (-8.0, 22.1)

Adjusted for gender, age, education, smoking status, drink status, SBP, UA, Scr, TC, TG, BUN, PLT, FBG, CRP. The stratified variable was not included in the model when stratifying by itself.

These findings emphasize the necessity of considering BMI and insulin resistance (IR) as an important risk factors in the development of strategies for preventing CVD in individuals with early-stage CKM syndrome, in conjunction with lipid control.

## 4 Discussion

This large prospective cohort study, based on data from CHARLS, is the first to investigate the association between HGI and the risk of CVD in early-stage CKM syndrome. Patients with CKM stages 0–3 who have not yet developed cardiovascular disease exhibit significant differences in clinical and metabolic characteristics across various groups. Individuals with high and rapidly increasing HGI levels demonstrate a greater prevalence of cardiovascular risk factors, leading to more complex comorbidities. HGI were the more important features than traditional risk factors such as FBG for predicting CVD. Additionally, we enhanced the understanding of the linear relationship between cumulative HGI and the incidence of CVD. TyG index, BMI and dyslipidemia showed potential mediating roles in the association between cumulative HGI and CVD. These findings further emphasize the crucial role of longitudinal monitoring of HGI in predicting CVD.

For patients with metabolic disorders, blood glucose levels are closely associated with the incidence and progression of vascular diseases. Hyperglycemia accelerates the non-enzymatic glycation of crucial proteins, causing the formation of glycated proteins. In addition to hemoglobin, other structural proteins are also susceptible to non-enzymatic glycation, leading to the formation of advanced glycation end products (AGEs) (29). AGEs trigger inflammatory signaling, enhance oxidative stress, and ultimately contribute to the development of atherosclerosis by damaging arterial endothelial cells and accelerating lipid oxidation (30). Although HbA1c remains a crucial tool for managing metabolic disorders, its limitations must be acknowledged. HGI could minimize clinical errors and optimize patient treatment, as a more personalized approach (19). Enhancing the management of HGI may not only help confirm the role of glycemic variability in the prevention and management of CKM syndrome, but also help make early lifestyle adjustments such as controlling glucose and cholesterol level.

This study is the first to investigate the association between cumulative HGI and CVD in a CKM syndrome population. Our results show that high cumulative level and rapidly growth of HGI is positively linked to the risk of CVD, consistent with previous research on single-measure HGI (31). In the Action to Control Cardiovascular Risk in Diabetes (ACCORD) trial, individual with high HGI values had a higher incidence of CVD (15). The ACCORD trial suggested that HGI could serve as a reference for adjusting treatment options to achieve improved CVD outcomes. Another study from Korea suggested that the development of CVD was significantly associated with baseline HGI in patients with type 2 diabetes (HR, 1.74; 95% CI, 1.08-2.81) (32). Due to the inter-individual variability of HGI, the large-scale studies are required to determine whether HGI can serve as a universality marker to assess CVD risk.

Asian populations bear a disproportionately high burden of diabetes and cardiovascular diseases. Due to genetic, dietary, and lifestyle factors, the relationship between HbA1c and blood glucose levels may exhibit unique characteristics in these populations (33). Longitudinal monitoring of the HGI can more accurately reflect glycemic variability and reduce the errors associated with relying solely on HbA1c. Our analysis also revealed this critical finding that the cumulative HGI demonstrated superior predictive capacity for CVD risk stratification in patients with CKM syndrome compared to conventional glucose metrics, including FBG. Some patients with diabetes exhibit higher postprandial glucose fluctuations and lower HbA1c levels, which may lead to an increased risk of inadequate glycemic control. By promoting the use of HGI monitoring, more precise glycemic assessment and personalized treatment strategies can be provided for Asian populations, thereby reducing the risk of cardiovascular diseases associated with CKM syndrome and minimizing potential harm caused by inaccurate glycemic evaluation and inappropriate therapeutic interventions.

Our findings revealed no statistical association between the HGI and CVD incidence during the ultra-early stages of CKM syndrome (stages 0 to 1). In individuals at stages 2 and 3 with elevated metabolic risk factors, the association between cumulative HGI and CVD incidence was more pronounced. This may be attributed to the fact that, metabolic disturbances in individuals may not yet reach the threshold level required to trigger cardiovascular risk in the early stages of CKM. Specifically, studies have shown that metabolic abnormalities, such as insulin resistance and chronic inflammation, need to accumulate to a certain extent before they can significantly impact vascular function and tissue damage (34). As an indicator reflecting long-term glycemic variability and hemoglobin glycation heterogeneity, HGI may lack sufficient sensitivity in the early stages when metabolic disturbances are relatively mild. This hypothesis is consistent with previous research, which indicates that the predictive ability of HbA1c and HGI is weaker in the prediabetic or early metabolic syndrome stages but becomes significantly enhanced as the disease progresses (35). During CKM stages 0-1, individuals may partially offset the vascular damage caused by glycemic fluctuations through compensatory mechanisms, such as enhanced insulin secretion or antioxidant capacity, thereby masking the predictive value of HGI. As the disease progresses to CKM stages 2-3, the decline in metabolic compensatory capacity allows the association between HGI and CVD risk to become more apparent. The latter stages of CKM are often accompanied by more pronounced chronic inflammation and oxidative stress, which may amplify the predictive role of HGI in CVD risk.

Reduced insulin sensitivity is considered as a significant risk factor for atherosclerotic disease (36), and serves as a central contributor to cardiovascular risk factors such as visceral obesity, atherogenic dyslipidemia, and hypertension, which frequently co-occur in individuals with metabolic disorders (37). In our study, an essential finding is that IR related indicators (TyG), BMI and dyslipidemia partially mediated the relationship between cumulative HGI and CVD. There is evidence that elevated HGI relate to the IR and increased risk of vascular atherosclerosis (38). The promotion of AGEs may alter insulin receptor signaling and impair glucose-uptake. In animal models, oral advanced AGEs have been shown to induce insulin resistance, contributing to metabolic disorders and lipid toxicity (39, 40).

IR can affect glucose metabolism through inflammatory factors, macrophage and adipocyte activation, and the renin–angiotensin–aldosterone system, can contribute to cardiac dysfunction and myocardial injury, ultimately leading to various cardiovascular diseases. Previous research has established that individuals with a metabolically healthy obesity phenotype face a higher risk of CVD compared to those with a metabolically healthy normal-weight profile (41). Marini et al. reported that elevated HGI may reflect the risk of metabolic disease associated with obesity (38). Our research further corroborates this finding. A study involving patients in American showed that BMI interacts with HGI, such that lower levels are associated with cardiovascular benefits (42). Regarding blood pressure, previous studies confirmed that BP status significantly modified the associations between cardiometabolic risk factors and CVD (43). HGI was found to be linked to arterial stiffening, independent of diabetes status (44).

With the progression of population aging, declining metabolic function and heightened chronic inflammatory states may amplify the impact of glycemic variability on CVD risk (45). Age is a non-modifiable risk factor for vascular diseases such as coronary heart disease and stroke, but metabolic abnormalities may represent critical modifiable targets for risk reduction (46). In age-stratified analyses, although the interaction P-value did not reach statistical significance, it is noteworthy that the strength of the association between HGI levels and CVD risk was more pronounced in the older age group compared to the middle-aged group. Based on the CHARLS database of older adults, this study demonstrates that HGI serves as a robust independent predictor of coronary heart disease risk. Given the accelerating global aging population, HGI is expected to play an increasingly significant role in risk stratification, offering valuable insights for early identification of high-risk individuals and the development of personalized intervention strategies.

To our knowledge, this is the first study to explore the mediating effects of these risk factors in the relationship between the HGI and adverse health outcomes. Although CRP was not identified as mediators in this association, this does not negate their potential relationship with HGI and CVD.

The potential limitations of this study should not be overlooked. First, the exclusion of individuals without FBG and HbA1c measurements led to the omission of a significant portion of the diabetic metabolic population, which may affect the findings. Second, CVD diagnoses in CHARLS were self-reported, and no further adjudication of CVD events was conducted. Third, HbA1c measurements in CHARLS were only taken at two time points (2012 and 2015), which may not adequately capture short-term fluctuations or the complete metabolic trajectory, potentially influencing the observed relationship. Fourthly, due to the limited variables available in the database, residual confounding from unmeasured inflammatory mediators or adipose-derived hormones might partially mediate the observed HGI-CVD association. Lastly, since HGI may have ethnic variability, multinational multicenter validation studies are necessary to establish its broader clinical applicability.

## 5 Conclusion

This study found that a rapidly increasing and high cumulative level of HGI was associated with an elevated risk of CVD progression in individuals with early-stage CKM syndrome. This association was partially mediated by BMI, TyG and dyslipidemia. HGI can serve as an independent predictor for assessing cardiovascular risk in patients with CKM syndrome. Our findings offer new insights into the potential relationship between blood glucose control, insulin resistance, lipid metabolism, and CVD in individuals with CKM syndrome. Clinicians should consider regular HGI monitoring to facilitate timely lifestyle interventions or therapy adjustments.

## Data Availability

The original contributions presented in the study are included in the article/**Supplementary Material**. Further inquiries can be directed to the corresponding author.
