# Supplementary material for: Association between hemoglobin glycation index and the risk of cardiovascular disease in early-stage cardiovascular-kidney-metabolic syndrome: evidence from the China health and retirement longitudinal study
Source: Front Endocrinol (Lausanne). 2025 May 8;16:1554032. doi: 10.3389/fendo.2025.1554032 (PMC12095031; doi:10.3389/fendo.2025.1554032)
Supplement: Supplementary file 1 [file DataSheet1.docx]

**Table S1 Interaction for the association between different terms and CVD**

| Subgroup | Value of HGI | | | | P for interaction | |
| --- | --- | --- | --- | --- | --- | --- |
|  | Class1 | Class2 | Class3 | Class4 |  |  |
| Gender |  |  |  |  | 0.492 | |
| Male | Reference | 0.90 (0.66 ~ 1.22) | 1.05 (0.86 ~ 1.27) | 1.05 (0.77 ~ 1.51) |  | |
| Female | Reference | 1.05 (0.80 ~ 1.38) | 1.21 (1.04 ~ 1.41) | 1.21 (0.96 ~ 1.51) |  | |
| Age |  |  |  |  | 0.499 | |
| <60 yr | Reference | 0.89 (0.68 ~ 1.16) | 1.10 (0.94 ~ 1.30) | 1.03 (0.81 ~ 1.31) |  | |
| ≥60 yr | Reference | 1.13 (0.82 ~ 1.55) | 1.14 (0.96 ~ 1.37) | 1.34 (1.03 ~ 1.74) |  | |
| Smoking |  |  |  |  | 0.800 | |
| Yes | Reference | 1.07 (0.83 ~ 1.38) | 1.08 (0.88 ~ 1.32) | 1.27 (0.93 ~ 1.74) |  | |
| No | Reference | 0.83 (0.59 ~ 1.16) | 1.17 (1.01 ~ 1.36) | 1.13 (0.91 ~ 1.41) |  | |
| Hypertension |  |  |  |  | 0.748 | |
| Yes | Reference | 0.92 (0.68 ~ 1.25) | 1.08 (0.91 ~ 1.29) | 1.06 (0.86 ~ 1.32) |  | |
| No | Reference | 1.03 (0.78 ~ 1.36) | 1.19 (1.01 ~ 1.40) | 1.35 (1.01 ~ 1.82) |  | |
| Dyslipidemia |  |  |  |  | 0.918 | |
| Yes | Reference | 0.93 (0.71 ~ 1.23) | 1.09 (0.93 ~ 1.27) | 1.30 (1.06 ~ 1.58) |  | |
| No | Reference | 1.10 (0.82 ~ 1.48) | 1.26 (1.06 ~ 1.51) | 0.77 (0.49 ~ 1.20) |  | |
| Diabetes |  |  |  |  | 0.842 | |
| Yes | Reference | 1.07 (0.98 ~ 1.16) | 1.17 (0.89 ~ 1.54) | 1.04 (0.83 ~ 1.31) |  | |
| No | Reference | 1.20 (1.08 ~ 1.33) | 1.13 (0.99 ~ 1.29) | 1.54 (1.13 ~ 2.10) |  | |
| CKM |  |  |  |  | 0.143 | |
| Stage 0 | Reference | 1.15 (0.55 ~ 2.43) | 1.37 (0.86 ~ 2.19) | None |  | |
| Stage 1 | Reference | 0.91 (0.53 ~ 1.57) | 1.09 (0.78 ~ 1.54) | 1.92 (0.77 ~ 4.75) |  | |
| Stage 2 | Reference | 1.00 (0.73 ~ 1.35) | 1.17 (0.99 ~ 1.38) | 0.97 (0.75 ~ 1.24) |  | |
| Stage 3 | Reference | 0.97 (0.68 ~ 1.40) | 1.01 (0.80 ~ 1.26) | 0.97 (0.75 ~ 1.24) |  | |
| Data are presented as OR (95% CI) unless indicated otherwise.  Adjusted for gender, age, education, smoking status, drink status, SBP, UA, Scr, TC, TG, BUN, PLT, FBG, CRP. The stratified variable was not included in the model when stratifying by itself.  OR, odd ratio; CI, confidence interval. | | | | | |  |

**
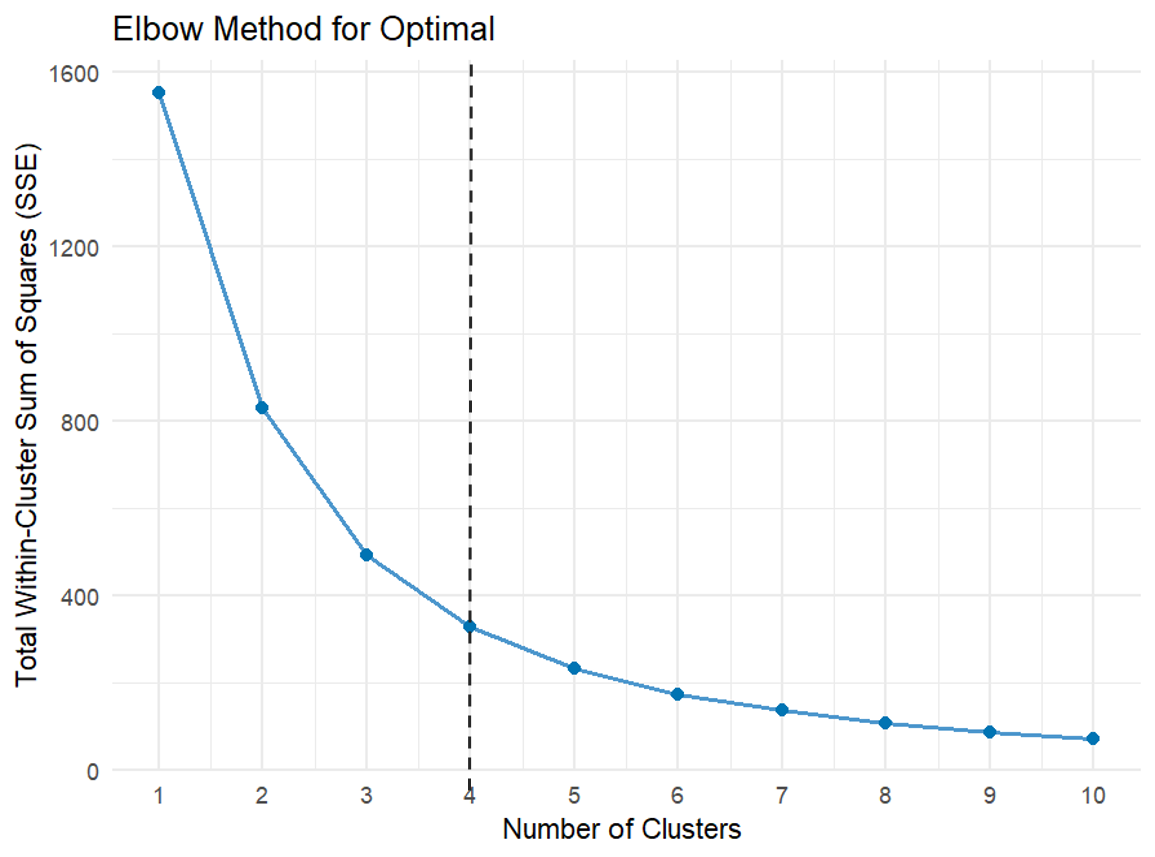
**

**Fig. S1** Elbow method to identify the appropriate number of clusters.

**
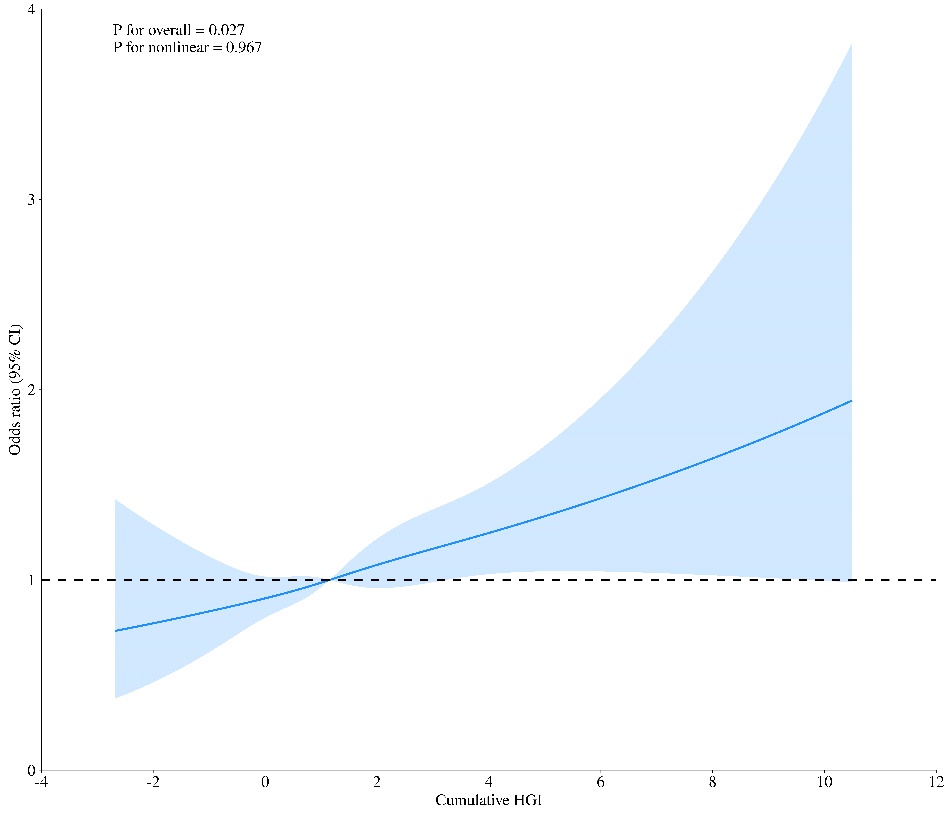
**

**Fig. S2** Association between the cumulative HGI and the incident of CVD in patients with early-stage CKM syndrome.
